# Blood Fatty Acids Profile in MIS-C Children

**DOI:** 10.3390/metabo11110721

**Published:** 2021-10-22

**Authors:** Elvira Verduci, Patrizia Risé, Elisabetta Di Profio, Laura Fiori, Sara Vizzuso, Dario Dilillo, Savina Mannarino, Elena Zoia, Valeria Calcaterra, Christian Pinna, Angelo Sala, Gianvincenzo Zuccotti

**Affiliations:** 1 Pediatric Department, “Vittore Buzzi” Children’s Hospital, 20154 Milan, Italy; elvira.verduci@unimi.it (E.V.); elisabetta.diprofio@unimi.it (E.D.P.); laura.fiori@asst-fbf-sacco.it (L.F.); sara.vizzuso@asst-fbf-sacco.it (S.V.); dario.dilillo@asst-fbf-sacco.it (D.D.); valeria.calcaterra@unipv.it (V.C.); gianvincenzo.zuccotti@unimi.it (G.Z.); 2 Department of Health Sciences, University of Milano, 20142 Milano, Italy; 3 Department of Pharmaceutical Sciences, University of Milan, 20133 Milano, Italy; patrizia.rise@unimi.it (P.R.); christian.pinna@unimi.it (C.P.); 4 Department of Animal Sciences for Health, Animal Production and Food Safety, University of Milan, 20133 Milan, Italy; 5 Pediatric Cardiology Unit, “Vittore Buzzi” Children’s Hospital, 20154 Milano, Italy; savina.mannarino@asst-fbf-sacco.it; 6 Anesthesia and Intensive Care Unit, “Vittore Buzzi” Children’s Hospital, 20154 Milano, Italy; elena.zoia@asst-fbf-sacco.it; 7 Pediatric and Adolescent Unit, Department of Internal Medicine, University of Pavia, 27100 Pavia, Italy; 8 IRIB, C.N.R., 90146 Palermo, Italy

**Keywords:** arachidonic acid, docosahexaenoic acid, eicosanoids, specialized pro-resolution mediators, inflammation

## Abstract

MIS-C (multisystem inflammatory syndrome in children) linked to SARS-CoV-2 infection, is a pathological state observed in subjects younger than 21 years old with evidence of either current SARS-CoV-2 infection or exposure within the 4 weeks prior to the onset of symptoms, the presence of documented fever, elevated markers of inflammation, at least two signs of multisystem involvement, and, finally, lack of an alternative diagnosis. They share with adult COVID-19 patients the presence of altered markers of inflammation, but unlike most adults the symptoms are not pulmonary but are affecting several organs. Lipid mediators arising from polyunsaturated fatty acids (PUFA) play an important role in the inflammatory response, with arachidonic acid-derived compounds, such as prostaglandins and leukotrienes, mainly pro-inflammatory and ω3 PUFA metabolites such as resolvins and protectins, showing anti-inflammatory and pro-resolution activities. In order to assess potential alterations of these FA, we evaluated the blood fatty acid profile of MIS-C children at admission to the hospital, together with biochemical, metabolic and clinical assessment. All the patients enrolled showed altered inflammatory parameters with fibrinogen, D-dimer, NT-proBNP, ferritin, aspartate aminotransferase (AST), C-reactive protein (CRP) and TrygIndex levels over the reference values in all the subjects under observation, while albumin and HDL-cholesterol resulted below the normal range. Interestingly, linoleic acid (LA), arachidonic acid (AA) and the ω3 PUFA docosahexaenoic acid (DHA) results were lower in our study when compared to relative amounts reported in the other studies, including from our own laboratory. This significant alteration is pointing out to a potential depletion of these PUFA as a result of the systemic inflammatory condition typical of these patients, suggesting that LA- and AA-derived metabolites may play a critical role in this pathological state, while ω3 PUFA-derived pro-resolution metabolites in these subjects may not be able to provide a timely, physiological counterbalance to the formation of pro-inflammatory lipid mediators. In conclusion, this observational study provides evidence of FA alterations in MIS-C children, suggesting a significant contribution of ω6 FA to the observed inflammatory state, and supporting a potential dietary intervention to restore an appropriate balance among the FAs capable of promoting the resolution of the observed inflammatory condition.

## 1. Introduction

During the COVID-19 pandemic, children appeared to be less affected than adults, and Severe Acute Respiratory Syndrome Coronavirus 2 (SARS-CoV-2) infections were in most cases asymptomatic or had mild symptoms [1,2,3]. However, from April 2020, in several western countries, a number of cases of children and adolescents with fever, hypotension, severe abdominal pain and cardiac dysfunction have been reported [4,5,6,7,8].

As stated by the Center of Diseases Control and Prevention (CDC), MIS-C (multisystem inflammatory syndrome in children) linked to SARS-CoV-2 infection requires patients to be less than 21 years old and to have evidence of either current SARS-CoV-2 infection or exposure within 4 weeks prior to the onset of symptoms, the presence of documented fever, elevated markers of inflammation, at least two signs of multisystem involvement, and, finally, lack of an alternative diagnosis (e.g., bacterial sepsis, toxic shock syndrome) [9]. While in Asian countries no cases of MIS-C were reported [10], in Western countries MIS-C represents a critical health condition associated with SARS-CoV-2 infection [11,12,13,14].

In COVID-19 adult patients, COVID-19 is typically associated with a significant degree of inflammation with an increase in interleukin 6 (IL6), C-reactive protein (CRP), fibrinogen, and erythrocyte sedimentation rate (ESR). High concentrations of D-dimer have been associated with increased mortality from COVID-19, and evidence in the literature suggest that COVID-19 coagulopathy is largely determined by the host inflammatory response, which in turn causes a pro-thrombotic status [15], leading to severe pulmonary manifestations similar to acute respiratory distress syndrome (ARDS) [16], even if non pulmonary organ damage was also present in the most severe cases [17].

In children with SARS-CoV-2 infections, the pulmonary manifestations are less severe than in adults, possibly because of a lower gene expression of the angiotensin converting enzyme (ACE)-2 receptor [18]. On the other hand, it has been suggested that MIS-C is a delayed immunological phenomenon associated with inflammation (stage-III hyperinflammation phase) following either symptomatic or asymptomatic COVID-19 infection [19], again pointing to a critical role of innate immunity and acute inflammation in the most severe forms of COVID-19.

The acute inflammatory response is associated with the production of several lipid mediators, including eicosanoids, a large family of compounds arising from the oxidative metabolism arachidonic acid known to play a significant role in various vascular and cellular events of the inflammatory response [20,21].

More recently, evidence has emerged that lipid mediators may also play a relevant role in the process leading to the physiological resolution of the inflammatory process, with a new genus of metabolites derived from ω3 polyunsaturated fatty acids (ω3-PUFA) being identified as possessing potent anti-inflammatory and pro-resolution properties [22]. In particular, docosahexaenoic acid (DHA) and eicosapentaenoic acid (EPA) metabolites resulting from the activity of several lipoxygenases have been collectively defined as specialized pro-resolving mediators (SPMs), a family which includes resolvins, protectins, maresins, and maresin-conjugate in tissue regeneration [23]. 

SPMs produced by the metabolism of ω3-PUFA have been reported to decrease the synthesis of pro-inflammatory mediators and neutrophil recruitment, activating at the same time macrophages with an anti-inflammatory phenotype (M2) and stimulating phagocytosis in a non-phlogistic manner [24]. Given their profile of activities, they clearly operate differently than currently available anti-inflammatory drugs, and do not show immunosuppressive effects [25].

Epidemiological data clearly show that obesity is a risk factor for SARS-CoV-2 infection and a BMI of 30 kg/m^2^ increases the risk of infection by 61% in adult patients. Interestingly, it has been hypothesized that a deficiency of SPMs in obese patients may promote severe outcomes during SARS-CoV-2 infection [26].

On the other hand, the use of the available enzyme inhibitor and receptor antagonists targeting 5-lipoxygenase metabolites of AA has been proposed as a strategy to limit the hyperinflammatory response to SARS-CoV-2 [27], in consideration of the evidence available about the presence of elevated leukotrienes in ARDS [28], and the efficacy of inhibitors and receptor antagonists in relevant preclinical models [29].

Taken together, a large body of evidence supports a potentially critical involvement of PUFA-derived lipid mediators in the evolution of the SARS-CoV-2 infection, and changes in serum lipid species have indeed been reported, with significantly decreased concentrations of AA and AA-containing phospholipids in adults and elderly (30–77 yo) COVID-19 patients [30].

In consideration of the potentially critical role played by PUFA metabolites in the evolution of SARC-CoV-2 infections in both young and adults, we carried out this observational study in order to assess fatty acid profiles in children and adolescents diagnosed with MIS-C while hospitalized at the Vittore Buzzi Children’s Hospital in Milan, Italy, during the pandemic.

## 2. Results

Table 1 summarizes the characteristics of the subjects at the study; children and adolescents after remission from COVID-19 were released from the intensive care unit (ICU) and moved to the pediatric unit at the Buzzi hospital, Milano, Italy. Of the 26 subjects studied, 81% were males; weight, BMI and BMI z score are reported. During ICU care, feeding was minimal, with a few cases requiring parenteral nutrition. The pharmacological regimen consisted in oral or i.v. corticosteroids (26 over 26), enoxaparin (22 over 26), ASA (5 over 26), and immunoglobulins (3 over 26).

The biochemical parameters reported in Table 2 showed that fibrinogen, D-dimer, NT-proBNP, ferritin, aspartate aminotransferase (AST), C-reactive protein (CRP) and TyG index levels were over the reference values in all the subjects (26/26 = 100%). An increase of TnT (68%), procalcitonin (96%), erythrocyte sedimentation rate (ESR) (61%) and IL-6 (89%) was also observed. Some parameters were below the normal values, as was the case for albumin and HDL cholesterol (100% of cases), as well as hemoglobin (72%).

The whole blood fatty acid profiles of these patients (Table 3) showed palmitic (16:0) and oleic (18:1 *n* − 9) acids as the most abundant FA, followed by linoleic (18:2 *n* − 6) and stearic (18:0) acids. Saturated FA accounted for 41.39 ± 2.39%, while polyunsaturated represented 24.13 ± 2.75 of total FA. The omega 3 index (OI3), calculated as described by Stark [36], ranged from 1.60% to 4.15% with a mean of 2.35%, with only one case where the O3I was higher than 4%, while all others cases were below the value of 3%.

We compared the relative quantities of some representative FA assessed in this study with the relative quantities reported by other studies (Table 4) in which the FA profile was obtained analyzing the whole blood of children and/or adolescents [37,38,39,40,41]. For studies examining differences in FA profile between control and pathological subjects, the values of the control group were considered. To better compare the data with those present in the literature, our subjects were also divided in those under (*n* = 14) or over 9 years old (*n* = 12). Levels of linoleic acid (LA), arachidonic acid (AA) and DHA were lower by an average of 38, 35 and 38%, respectively, in MIS-C subjects when compared to values reported for children of similar age in these other studies, including the results of a study carried out in our own laboratory [37]. The EPA that resulted was in line with other values reported, whereas α-linolenic acid (ALA) was higher mainly because of values observed in the group >9 yo.

The possible correlations between relative FA amounts and the inflammatory parameters were also investigated (Table 5), but only a statistically significant positive correlation between ALA and CRP was present, with a Spearman correlation coefficient of 0.508 (*p* < 0.05). It must be noted that samples for the evaluation of inflammatory parameters and for FA analysis were drawn 5–7 days apart from each other.

## 3. Discussion

On May 2020, the Centers for Disease Control detailed the criteria of MIS-C diagnosis, that, together with a previous or current SARS-CoV-2 infection and two signs of multisystem involvement, also includes the presence of altered markers of inflammation. While sharing common features with Kawasaki Disease, toxic shock syndrome, and secondary hemophagocytic lymphohistiocytosis/macrophage activation syndrome [19], MIS-C importantly shares with COVID-19 the presence of laboratory evidence of inflammation, and it has been hypothesized that viral replication affects the production and release of inflammatory mediators, leading to a severe inflammatory reaction both in MIS-C and in COVID-19. Elevated concentrations of cytokines including IL-6, as well as increased CRP and D-dimer concentrations, are common in severe COVID-19 patients [42,43], and the uncontrolled inflammatory response has been deemed responsible for the most severe forms of COVID-19. 

Together with cytokines, lipid mediators also play a critical role in the physiological evolution of the acute inflammatory reaction: oxygenated metabolites arising from ω6 PUFA may participate in both the propagation and the resolution of the inflammatory response [44], but they mainly exert potent proinflammatory and prothrombotic activities. Indeed, specifically targeting the production and the activity of AA-derived leukotrienes has been proposed as a novel approach to modulate the hyperinflammatory state in COVID-19 subjects [27], and increased concentrations of LA-derived leukotoxin diols were detected in the plasma of severe COVID-19 patients [45]. On the other hand, ω3 PUFA (i.e., EPA and DHA) are the substrates responsible for the formation of a new genus of anti-inflammatory and pro-resolution lipid mediators collectively named specialized pro-resolving mediators (SPM) [23,24]. While plasmatic concentrations of SPM did not change upon LPS challenge in healthy volunteers [46], recent reports of their rapid metabolism suggests that plasmatic concentrations may not be predictive of the actual production in vivo [47], as is well known for eicosanoids. Based on their potent biological activities, SPMs may therefore play a critical role in the physiological resolution of the acute inflammatory response, and may be able to counteract the hyperinflammatory status observed not only in severe COVID-19 subjects [26,48], but also in MIS-C children.

In line with previously published results [19,49], clinical and biochemical parameters in the observed subjects support the diagnosis of MIS-C. They have had COVID-19 infection, and present multiorgan system failure, with persistently elevated levels of ferritin, CRP, fibrinogen, IL-6, procalcitonin, and increased ESR, all typical markers of an ongoing inflammatory condition. The presence of glucose metabolism alterations also observed in this sample of children with MIS-C, such as elevated TyG index, suggests that a bidirectional relationship between COVID-19 and glycemic impairment could not be excluded. Indeed, hyperglycemia and glycemic fluctuations could be caused by the inflammatory cascade triggered by SARS-CoV-2 in the pancreas, and the potentially impaired β-cell function associated with the SARS-CoV-2 entry through the ACE2 receptor [49].

The analysis of the whole blood fatty acid profile revealed relative amounts of ω6 PUFA in MIS-C subjects lower than those reported in literature, both for LA and AA. The same holds true upon subdivision of our group of subjects into children <9 and >9 years old, in order to facilitate a direct comparison with literature data relative to children of different age ranges. The levels of ω3 PUFA ALA and EPA were substantially in line with those present in the literature, whereas DHA levels were also lower [37,38,39,40,41]. It is important to note that the FA values observed in normal children by our group and reported in [37] referred to normal weight or overweight children, in line with the characteristics of the subjects observed in this study, whereas Bonafini et al. reported the data of normal weight children only [39]. A few studies suggested the occurrence of an altered FA profile in obese children [50,51], but the statistical analyses of a large number of data processed for the IDEFICS study reported only minor differences in FA levels if obese children were included [52].

These data are in agreement with changes observed in adult severe COVID-19 subjects: serum metabolomic/lipidomic analysis, carried out in a population aged 20–70 years showed not only that AA-containing phosphatidylcholine (16:0–20:4) and AA levels decrease with the severity of the disease [30], but also an increase in lysophospholipids reflecting PLA2 activity leading to significant AA mobilization from phospholipids, as observed in other pulmonary infections [53].

The lower levels of AA in children with MIS-C may therefore be the result of massive release from phospholipid storage followed by metabolic conversion into pro-inflammatory lipid mediators. Increased formation of SPM may also be occurring, as supported by the observed decrease in DHA, but in these subjects the associated formation of SPM did not appear to be enough to stave off the hyperinflammatory state confirmed by the clinical conditions and the altered biochemistry parameters reported.

LA is formally the biological precursor of AA, but only about 2% is converted into AA in humans [54], and in spite of being an essential FA, its current intake with the diet is much higher than the essential levels of linoleate, so that actual depletion is substantially impossible in the absence of an inborn error of metabolism [55]. The decrease of this FA observed in MIS-C children may find a cause very similar to that of AA. In fact, LA is the substrate for the CYP450 enzymes, including CYP2J2, CYP2C8, CYP2C9, and CYP1A1, leading to the formation of linoleic epoxides 9,10-epoxyoctadecenoic acid (9,10-EpOME) and 12,13-epoxyoctadecenoic acid (12,13-EpOME) known as leukotoxin and isoleukotoxin [56]. These epoxides are then metabolized by the soluble epoxide hydrolases (sEH) into the dihydroxyderivatives 9,10-DiHOME and 12,13-DiHOME, with the former known as a major contributor to pulmonary toxicity in acute respiratory distress syndrome (ARDS) [57]. Hospitalized COVID-19 patients with severe pulmonary involvement presented increased plasmatic amounts of regioisomeric leukotaxin diols [45], suggesting that the formation of these LA metabolites could be associated with the most severe forms of COVID-19. Again, the altered FA profile observed in MIS-C patients seems to point to a massive formation of pro-inflammatory lipid mediators potentially leading to decreased relative amounts of their precursor FAs.

No statistically significant correlation was found between inflammation markers and relative amounts of relevant FA (i.e., LA, AA, EPA and DHA), while a direct correlation between relative ALA quantities and CRP concentration was present. ALA has been shown to represent the precursor of ω3 PUFA, but its conversion is unreliable in humans [58], and its association with CRP may even indicate that a restricted conversion from ALA to DHA may be leading to a decreased formation of pre-resolution SPM and increased inflammatory biomarker(s). It must be noted that sampling for the evaluation of inflammatory parameters and for FA analysis was carried out at 5–7 day intervals.

Inverse associations between the LA, total ω6 PUFA, EPA/AA and DHA/AA ratios and hs-CRP were reported in a study of children [59], with differences emerging between boys and girls. Nevertheless, in that work, the population examined was composed of healthy young children in the absence of pathological states and therefore with very low CRP concentrations which were in some cases not detectable. This could explain the differences with our data, where the average CRP was in the range of 200 mg/L. 

O3I, a recognized good biomarker of ω3 PUFA status, has also been reported as being inversely associated with some inflammatory biomarkers such as IL-6 and CRP [60]. In our study O3I did not associate with IL-6, CRP or ESR, but it must be stressed that typical association studies, including González-Gil et al. and Fontes et al., have been examining populations of subjects in “normal” conditions, i.e., in the absence of an acute inflammatory state such as the one reported in our study. A pilot study investigating the possible association between O3I and risk of death in COVID-19 patients (*n* = 100), found that patients with a O3I equal or greater than 5.7% were at lower risk of death (about 75%) compared to patients with an O3I lower than that value [61]. In light of our interpretation of the ω6 and ω3 PUFA values observed in MIS-C children, the O3I values obtained in hospitalized COVID-19 subjects may reflect the severity of the inflammatory status, with higher values in milder cases, and vice-versa, resulting from the use of EPA and, in particular, DHA to synthesize SPM in order to counterbalance the massive AA-derived pro-inflammatory metabolite formation resulting from SARS-CoV-2 infection.

The western, modern diet is a diet rich in ω6 PUFA with low levels of ω3 PUFA, a condition that causes a “chronic state of low-grade inflammation” [62], and therefore the baseline status of ω6 and ω3 PUFA, as has been proposed for cardiovascular, neurodegenerative, or autoimmune diseases with a critical inflammatory component, may be also of relevance for COVID-19 as well as for MIS-C subjects with respect to their ability to cope with the hyperinflammatory state induced by SAR-CoV-2. 

Based on the potential anti-inflammatory and pro-resolution effects of ω3 PUFA and their endogenously generated metabolites (SPM) [22,23,24], it has been hypothesized that ω3 PUFA supplementation could be beneficial in COVID-19 patients [63], and parenteral infusion of pure fish oils for hospitalized patients with COVID 19 has also been proposed to attenuate respiratory failure and to reduce infection, sepsis rate and hospital length of stay, due to its proven clinical efficacy in patients with ARDS [64]. Interestingly, the results of randomized, double-blinded clinical trials have indeed recently appeared in the literature, showing that ω3 PUFA supplementation with one capsule of 1000 mg ω3 daily containing 400 mg EPA and 200 mg DHA for 14 days improved the levels of many parameters of respiratory and renal function as well as the survival in critically ill adult COVID-19 patients [65]. 

Our work, to our knowledge, is the first reporting the whole blood FA profile of children with MIS-C, showing a lipidic dysregulation similar to that observed in COVID-19 adults, with decreased levels of LA, AA and DHA as a possible result of increased metabolic use for the formation of the lipid mediators participating in the hyperinflammatory inflammatory status observed. This observational study comes with several limitations, including the relatively small number of subjects enrolled, resulting in the imbalance between the number of boys and girls, while the absence of a control group is partially compensated by the fact that the published data relative to FA profiles in children used to compare the values observed in this study with normal values were indeed generated in our laboratory, and therefore used the same analytical approach. The observation of a potentially significant contribution of lipid mediators to the MIS-C phenotype also suggests that a possible dietary intervention could aim at tilting the balance between ω6 and ω3 PUFA, helping steer patients toward the final formation of anti-inflammatory, pro-resolution lipid mediators. Nevertheless, proving that a dietary intervention is beneficial in these patients will be difficult, in consideration of the small number of children affected. To date, there are no clinical trials enrolling children and adolescents with SARS-CoV-2 infection aimed at assessing the effects of DHA or other ω3 PUFA supplementation. The promising activities of ω3 PUFA notwithstanding, more experimental, randomized control trials and epidemiological research is warranted before recommending this approach.

## 4. Materials and Methods

### 4.1. Subjects

A group of 26 children and adolescents with MIS-C, as defined according to the CDC classification [9], were enrolled at the Pediatric Department of Children’s Hospital Vittore Buzzi in Milan, Italy, between 1 December 2020 and 12 February 2021.

For all patients, a clinical and biochemical assessment was recorded on admission. Moreover, anthropometric measurements were collected at time of admission and before hospital discharge. 

After 5–7 days from admission, a drop of blood was collected on Guthrie Test paper from each patient and stored in a refrigerator until analysis as described below. At this time, the drug therapy of children was also recorded. 

The study was conducted according to the guidelines of the Declaration of Helsinki and approved by the Institutional Review Board of the hospital (protocol number 2021/ST/004). Children’s caregivers gave their written consent for inclusion after being informed about the nature of the study.

### 4.2. Anthropometric and Blood Measurements

Physical examination included anthropometric measurements of weight and height, Body Mass Index (BMI) calculation and evaluation of the pubertal stage was made for each patient. Weight and height were measured using a mechanical column scale with altimeter (Seca 711 and Seca 220), arm and waist circumferences were measured with atape measure (Seca 201) and tricipital skin-folds were measured using a caliper (Holtain 610). BMI (kg/m2) and BMI Z-SCORE were established according to CDC growth chart reference values [31,32].

The diagnostic procedure for confirming the MIS-C diagnosis involved a complete blood count and measurements of C-reactive protein (CRP), procalcitonin, ferritin, cardiac troponin T (cTnT), N-terminal pro-brain natriuretic peptide (NT-proBNP), coagulative parameters, creatine kinase, electrolytes, and interleukin-6 (IL-6). These measures were compared to our clinical laboratory’s normal range values. 

Additionally, the metabolic profile including total and high-density lipoprotein cholesterol (HDL), fasting plasma glucose (FPG), insulin and triglycerides (TG) was acquired from a blood sample obtained in a fasting state between 8:30 and 9:00 a.m. Insulin was measured using the electrochemiluminescence immunoassay (ELCIA) method.

The triglyceride–glucose (TyG) index as a surrogate for insulin resistance was calculated as [ln(fasting triglycerides (mg/dL) × fasting plasma glucose (mg/dL)/2)] [66,67]; the cutoff point for pathological IR was set at 7.88 [35,68].

Guthrie Test paper was used to collect whole blood for the analysis of fatty acids (FA) 5–7 days from admission, during the acute phase of the disease, for each patient. 

### 4.3. Fatty Acid Analysis

The FA profile was evaluated in a drop of blood collected on a Guthrie paper embedded with butylated hydroxy toluene (BHT) as antioxidant. After direct transmethylation, FA methyl esters were analyzed by gas chromatography using a GC-2100 (Shimadzu Italia S.r.l., Milano, Italy) equipped with a 15 m capillary column (DBB Agilent), PTV injector and FID detection [69,70]. Relative percentages were used to report 23 FA; total saturated FA (SAT), monounsaturated FA (MUFA) and PUFA were also reported. In addition, the omega 3 index (O3I) was calculated in accordance with Stark et al. [36]. 

### 4.4. Statistical Analysis

A descriptive statistical analysis was performed using IBM SPSS statistics version 27, whereas Spearman’s correlation coefficients were estimated for biochemical parameters and FA.

## Figures and Tables

**Table 1:** Anthropometric data of MIS-C children at hospital admission. Values are expressed as mean ± standard deviation (SD).

	Mean ± SD	Reference Values
N of subjects	26	
M/F	21/5	
Age (mo)	110.79 ± 50.29	
Height (cm)	135.86 ± 24.24	
Weight (kg)	36.35 ± 16.26	
BMI (kg/m^2^)	18.58 ± 3.19	
BMI z score [31]	0.48 ± 1.00	±2 z score obesity and malnutrition (CDC)
Arm circ (cm) [32]	21.16 ± 4.27	<5° or >90° pc (NANHES III)
Waist circ (cm) [32]	66.55 ± 10.48	<5° or >90° pc (NANHES III)
Triceps skinfold (mm) [32]	15.05 ± 7.80	<5° or >90° pc (NANHES III)

**Table 2:** Biochemical data of MIS-C children at hospital admission. Values are expressed as median and 25th–75th percentile. The % of subjects with values outside of the reference intervals is also reported.

Laboratory Markers of Inflammation	Median (25th–75th Percentile)	Reference Values	% Subjects Outside of the Ref Values
Fibrinogen [33]	7 (6.4–7)	<4 g/L	100
D-dimer [33]	2734.5 (1956–4432.5)	<500 µg/L	100
CRP	236.5 (109–291.4)	≤10 mg/L	100
ESR	38 (23–69)	≤30 mm	61
Procalcitonine [33]	6.9 (2.3–24)	<0.5 µg/L	96
IL-6 [33]	75 (10–253)	≤7 ng/L	89
LDH [33]	255 (221.2–287)	180–360 U/L	
Ferritin [33]	875.5 (414.8–1960.2)	<300 µg/L	100
Albumin	25.5 (22.5–29)	35–50 g/L	100
**Biochemistry**			
Hb [33]	11 (9.2–11.6)	11.5–15.5 g/dL	72
Leukocytes	8.4 (5.1–14.2)	4.5–10 × 10^9^/L	40
Platelets	156 (111.5–210.5)	M 155–320, F 169–359 × 10^9^/L	44
INR	1.3 (1.2–1.4)	<1.2	76
Ratio aPTT	1.4 (1.2–1.4)	0.84–1.16	86
TnT	47.5 (16–81.5)	≤15 ng/L	81
NT-proBNP	6619 (2621–14,594)	<450 ng/L	100
Creatinine	0.5 (0.4–0.7)	0.15–0.75 mg/dL	
Urea	29 (16.5–44)	19–50 mg/dL	
CK	68 (35.8–147.2)	M 47–322 U/L; F 29–201 U/L	
AST	57 (43–88.5)	11–34 U/L	100
ALT	31 (16.5–63.2)	M ≤ 49U/L, F ≤ 33 U/L	32
GGT	26.5 (20–56.2)	M 12–68 U/L, F 6–40 U/L	20
Na^+^ [33]	132 (130–135)	135–145 mmol/L	16
K^+^ [33]	3.5 (3–4)	3.5–5 mmol/L	52
TSH [33]	2.3 (1.3–3)	0.5–4.2 mIU/L	
fT3 [33]	2.7 (2–3.6)	3.5–6.3 pmol/L	80
fT4 [33]	12.2 (11.1–14.3)	9–19.3 pmol/L	
**Blood lipid and glucose metabolism**			
Total cholesterol [33]	120 (85–164)	<170 mg/dL	12
HDL-cholesterol [33]	16 (7–25.2)	>45 mg/dL	100
Triglycerides [33]	190 (124–303.2)	<75 mg/dL 0–9 yo <90 mg/dL age 10–19 yo	52
TyG Index [34]	9.2 (8.8–9.7)	<7.88	100
Glucose [35]	110.5 (95.5–125.2)	70–110 mg/dL	42
HbA1c [35]	33 (32–34.8)	≤39 mmol/mol	

**Table 3:** Whole blood fatty acid profile in children (*n* = 26) with MIS-C. Data are expressed as mean ± standard deviation (SD) of FA of the relative percentage (*weight/weight*) of all FA considered, analyzed as described in Methods.

FA	% *w*/*w* ± SD	(Min–Max)
16:0	27.99 ± 1.68	(24.65–30.90)
18:0	10.16 ± 1.26	(7.61–13.03)
20:0	0.41 ± 0.10	(0.26–0.62)
22:0	1.05 ± 0.22	(0.67–1.45)
24:0	1.75 ± 0.57	(1.13–3.05)
16:1	3.32 ± 1.09	(1.58–6.44)
18:1 *n* − 9	26.87 ± 3.18	(20.30–32.72)
18:1 *n* − 7	1.82 ± 0.52	(1.18–3.94)
20:1	0.19 ± 0.10	(0.05–0.63)
22:1	0.09 ± 0.04	(0.02–0.18)
24:1	2.21 ± 0.64	(1.55–3.64)
20:3 *n* − 9	0.15 ± 0.10	(0.04–0.44)
18:2 *n* − 6	12.39 ± 2.28	(8.29–16.99)
18:3 *n* − 6	0.64 ± 0.36	(0.09–1.78)
20:3 *n* − 6	1.09 ± 0.26	(067–1.74)
20:4 *n* − 6	6.38 ± 1.05	(4.86–8.54)
22:4 *n* − 6	0.81 ± 0.21	(0.54–1.35)
22:5 *n* − 6	0.42 ± 0.18	(0.27–1.14)
18:3 *n* − 3	0.22 ± 0.10	(0.09–0.48)
20:5 *n* − 3	0.34 ± 0.09	(0.17–0.49)
22:5 *n* − 3	0.48 ± 0.09	(0.28–0.69)
22:6 *n* − 3	1.21 ± 0.43	(0.39–2.89)
SAT	41.38 ± 2.39	(37.42–46.37)
MONO	34.50 ± 3.19	(25.88–40.09)
POLY	24.13 ± 2.75	(18.36–31.33)

FA: fatty acids; SD: standard deviation; SAT: saturated FA; MONO: monounsaturated FA; POLY: polyunsaturated FA.

**Table 4:** Relative quantities of relevant fatty acids observed in our study in comparison with data present in the literature. Fatty acid data are expressed as mean ± standard deviation of the relative percentage values, or, in the case of [40], the range of percentage values. For studies examining differences in the FA profile between control and pathological subjects, the values of the control group were considered.

Author (Reference)	Present Study	Risé [37]	Crippa [38]	Bonafini [39]	Van der Wurff [40]	Ryan [41]
Age (yo)	3–18	<9	7–14	7–9	13–15	4
% LA	12.39 ± 2.28		22.54 ± 2.45			
<9 yo	12.72 ± 2.43	17.67 ± 1.92		19.9 ± 2.32		
>9 yo	11.99 ± 2.13					
% AA	6.38 ± 1.05		10.10 ± 0.92			
<9 yo	6.14 ± 1.09	8.33 ± 1.04		12.21 ± 1.67		7.50 ± 1.89
>9 yo	6.66 ± 0.97				11.01–11.33	
% ALA	0.22 ± 0.10					
<9 yo	0.18 ± 0.10	0.15 ± 0.05		0.16 ± 0.08		
>9 yo	0.27 ± 0.09					
% EPA	0.34 ± 0.09		1.13 ± 0.45			
<9 yo	0.32 ± 0.09	0.23 ± 0.08		0.30 ± 0.17		0.30 ± 0.39
>9 yo	0.36 ± 0.08				0.34–0.42	
% DHA	1.20 ± 0.43		1.93 ± 0.53			
<9 yo	1.08 ± 0.28	1.40 ± 0.37		2.92 ± 0.76		1.00 ± 0.34
>9 yo	1.35 ± 0.53				2.49–2.63	

AA: arachidonic acid; ALA: α-linolenic acid; DHA: docosahexaenoic acid; EPA: eicosapentaenoic acid; LA: linoleic acid.

**Table 5:** Correlations between some relevant fatty acids and biochemical parameters of inflammation. Statistical significance was estimated using Spearman’s non parametric correlation coefficient. * *p* < 0.05.

	LA	AA	ALA	EPA	DHA	O3I
**CRP**	−0.023	−0.036	0.508 *	0.048	0.149	0.246
**IL-6**	−0.236	−0.378	−0.092	−0.097	−0.174	−0.117
**ESR**	0.466	0.054	−0.161	−0.140	0	−0.046

AA: arachidonic acid; ALA: α-linolenic acid; CRP: C-reactive protein; DHA: docosahexaenoic acid; EPA: eicosapentaenoic acid; ESR: erythrocyte sedimentation rate; IL-6: interleukin-6; LA: linoleic acid; O3I: omega 3 index.

## Data Availability

The data presented in this study are available on request from the corresponding author. The data are not publicly available for privacy.
